# The importance of the urinary output criterion for the detection and prognostic meaning of AKI

**DOI:** 10.1038/s41598-021-90646-0

**Published:** 2021-05-27

**Authors:** Jill Vanmassenhove, Johan Steen, Stijn Vansteelandt, Pawel Morzywolek, Eric Hoste, Johan Decruyenaere, Dominique Benoit, Wim Van Biesen

**Affiliations:** 1 Renal Department, Gent University Hospital 0K12IA, C. Heymanslaan 10, 9000 Ghent, Belgium; 2 Department of Applied Mathematics, Computer Science and Statistics, Ghent University, Ghent, Belgium; 3 Centre for Justifiable Digital Healthcare, Ghent University Hospital, Ghent, Belgium; 4 Department of Medical Statistics, London School of Hygiene and Tropical Medicine, London, UK; 5 Department of Intensive Care Medicine, Ghent University Hospital, Ghent, Belgium

**Keywords:** Nephrology, Kidney diseases, Acute kidney injury, Risk factors

## Abstract

Most reports on AKI claim to use KDIGO guidelines but fail to include the urinary output (UO) criterion in their definition of AKI. We postulated that ignoring UO alters the incidence of AKI, may delay diagnosis of AKI, and leads to underestimation of the association between AKI and ICU mortality. Using routinely collected data of adult patients admitted to an intensive care unit (ICU), we retrospectively classified patients according to whether and when they would be diagnosed with KDIGO AKI stage ≥ 2 based on baseline serum creatinine (Screa) and/or urinary output (UO) criterion. As outcomes, we assessed incidence of AKI and association with ICU mortality. In 13,403 ICU admissions (62.2% male, 60.8 ± 16.8 years, SOFA 7.0 ± 4.1), incidence of KDIGO AKI stage ≥ 2 was 13.2% when based only the SCrea criterion, 34.3% when based only the UO criterion, and 38.7% when based on both criteria. By ignoring the UO criterion, 66% of AKI cases were missed and 13% had a delayed diagnosis. The cause-specific hazard ratios of ICU mortality associated with KDIGO AKI stage ≥ 2 diagnosis based on only the SCrea criterion, only the UO criterion and based on both criteria were 2.11 (95% CI 1.85–2.42), 3.21 (2.79–3.69) and 2.85 (95% CI 2.43–3.34), respectively. Ignoring UO in the diagnosis of KDIGO AKI stage ≥ 2 decreases sensitivity, may lead to delayed diagnosis and results in underestimation of KDIGO AKI stage ≥ 2 associated mortality.

## Introduction

Acute Kidney Injury (AKI) is an important clinical condition with substantial impact on morbidity and mortality. The lack of a common universally accepted definition for AKI has for a long time hampered research and progress^1^. As a synthesis of different initiatives^2–6^, the KDIGO criteria became the standard for defining AKI. However, most reports, studies and automated detection and risk prediction tools still use a truncated version of the KDIGO definition based only on a serum creatinine (SCrea) criterion and neglect the urinary output (UO) criterion to define their ground truth definition for AKI^7–13^. In addition, just as is the case for the selection of a baseline creatinine measure, UO criteria can be implemented in different ways^14^. As a result, despite the unified definition as proposed by KDIGO, substantial cross-study and cross-model differences remain in what exactly is reported or predicted as AKI^7,8,15–18^.

So far, the problem of variable meanings of the AKI label was deemed to be only relevant for epidemiology^19^. It could however also have an important impact for the correct interpretation and transportability of clinical trial results. In addition, with the advent of electronic health records within the last decade, algorithms to automatically diagnose and predict AKI will be implemented in the everyday care of individual patients^20,21^. Such automated detection algorithms or risk prediction models could be used in early recognition and timely management of AKI^22,23^ and are considered a highly promising breakthrough for medicine^24,25^. These algorithms and models, however, are trained on databases which may contain different ‘ground truth’ labels of AKI, all loosely defined within the confines of the KDIGO guidelines, but mostly neglecting the UO criterion. This is problematic for interpretation and external validation because the gold standard against which their performance is assessed may be either different or simply missing in external databases, potentially leading to unsensible or misleading performance assessments.

Within the confines of the KDIGO definition, it can be hypothesized that omitting the UO criterion in the applied definition of AKI substantially downplays the importance of the AKI label if 1/neglecting UO in the ground truth substantially alters the incidence of AKI and leads to missed cases; 2/ neglecting the UO criterion often delays AKI diagnosis; 3/ the UO criterion has an independent association with ICU mortality and may therefore have added prognostic value compared to single use of the SCrea criterion. Several studies have provided evidence that is mostly in line with this hypothesis^15,26,27^. However, none of these studies has adequately accounted for the time dynamics of AKI diagnosis or documented the number of potentially missed cases or delayed diagnoses induced by ignoring the UO criterion as they evolve over time. Failure to account for the timing of diagnosis may moreover have introduced immortal time bias in assessing the association with adverse outcomes, which may have led to underestimation of these associations^28^. In this paper, we address these shortcomings with the aim to add to and strengthen the evidence base of the aforementioned hypotheses.

## Methods

All 52 beds of the Gent University Hospital (tertiary care) adult intensive care units (ICU) dispose of a commercially available Intensive Care Information System (ICIS) (Centricity Critical Care, GE Healthcare, Germany) to collect granular longitudinal patient data in real time. All data from monitors, ventilators, pumps, radiology results and laboratory results and administered medication are automatically uploaded in the system. In addition, a wide variety of pre-defined items are routinely entered manually by medical staff.

For all patients (aged 16 or older) admitted to our ICU from January 2013 to December 2017, we have included their first ICU episode during this study period for analysis. Raw data relevant for our study were extracted in a pseudonymized way from the ICIS database. Daily serum creatinine (SCrea) and all urinary output (UO) measurements during ICU stay were extracted from the ICIS, along with their corresponding sample times. For SCrea, this information originates from the lab information system directly linked to the ICIS. The study data set was complemented with all available SCrea values available to the lab information system up to 365 days before ICU admission. UO was periodically measured and entered into the ICIS by nursing staff who recorded the reading, mostly from a drainage container of a urinary catheter or from spontaneous urination in a scaled recipient. In addition, for each included patient, time from ICU admission to discharge, vital status at ICU discharge, gender, age, weight, comorbidities and Sequential Organ Failure Assessment (SOFA) score on the day of ICU admission were extracted.

Ethical approval was obtained from the Ethical Committee of Ghent University Hospital (EC nr 201-0705) and informed consent was obtained according to applicable regulations. All methods were performed in accordance with the relevant guidelines and regulations and the declaration of Helsinki.

The KDIGO criteria for AKI stage ≥ 2 diagnosis hinge on two different parameters: 1/ absolute or relative increase in SCrea as compared to a baseline measurement (Screa criterion) and 2/ oliguria during a 12-h period (UO criterion). As primary endpoints we considered (cumulative) incidence of AKI stage ≥ 2 diagnosis during ICU stay as defined by the KDIGO guidelines^29^, based on either criterion, or both, and the association between diagnosis by each of these criteria and ICU mortality, as a crude reflection of their prognostic value.

We intended to mimic a system in which automated computer code incorporated in an intensive care information system is applied to detect or alert for AKI in real-time. For each patient, we assessed whether and when any Screa measurement was found during their ICU stay that either exceeded 4.0 mg/dl or indicated a > twofold increase relative to one of the following baseline Screa measurements, selected in the order listed below, according to availability:

- *Screa-1* A baseline Screa measurement as manually entered in the ICIS by the treating physician at ICU admission. Physicians have to enter a (documented) Screa measurement before the current hospitalization and no older than 365 days or, if not available, the lowest measurement of the current hospitalization.
- *Screa-2* The lowest available pre-ICU measurement up to 365 days before ICU admission as extracted from the lab information system. This could be a value from before or during the index hospitalisation but before admission to ICU.
- *Screa-3* A back-calculated baseline Screa using the simplified 4-variable Modification of Diet in Renal Disease (MDRD) Study equation assuming an estimated glomerular filtration rate (eGFR) of 75 ml/min/1.73 m^2^ for every patient^30^.

In the interest of space, we only provide results for automated AKI diagnosis based on implementation of the Screa criterion relative to a baseline selected by this stepwise approach (henceforth labelled Screa). Readers are referred to the supplemental material for results relative to each of these listed and two additional baseline measurements separately.

To identify patients with AKI stage ≥ 2 based on the UO criterion, UO measurements were first converted to ml/kg/h. Patients’ weight was entered into the ICIS by nursing staff based on recent weight registrations when available in the hospital information system, self-report from the patient, from relatives, or an estimated clinical guess. In line with the KDIGO guidelines, we assessed for each patient whether and when any UO registration was found during their ICU stay at which

- *UO-1* total UO during the last 12-h period was ≤ 6 ml/kg
- *UO-2* total UO during each of the last 12 consecutive 1-h periods was ≤ 0.5 ml/kg

UO was calculated in X-hour time windows according to the rules described in Supplementary Material S1 (section “Data preprocessing”).

In addition, to assess the relative impact of ignoring either of these criteria, two composite definitions of AKI stage ≥ 2 were postulated:

- *Screa-UO-1* which indicates whether a patient is diagnosed based on either the Screa criterion or the UO-1 criterion (as defined above)
- *Screa-UO-2* which indicates whether a patient is diagnosed based on either the Screa criterion or the UO-2 criterion (as defined above).

Cumulative incidence curves were obtained to assess the dynamics of how the number of incident AKI stage ≥ 2 cases, as diagnosed by each of the aforementioned criteria, accumulates over time. Comparison of these curves enables to retrospectively assess, at each time point, the percentage of cases that were either missed or diagnosed with delay by ignoring either the SCrea criterion or the UO criteria. Additional details on calculation of number of cases missed or diagnosed with delay can be found in Supplementary Material S1 (section “Cumulative incidence curves”).

The associations between diagnosis by each of the aforementioned criteria and ICU mortality, as captured by cause-specific hazard ratios comparing patient time at risk of ICU mortality with versus without diagnosis, were estimated using a series of extended Cox proportional hazards models for time from admission to ICU death. By including AKI stage ≥ 2 diagnosis as a time-varying rather than a time-fixed covariate (coded 1 from the time of AKI diagnosis and 0 otherwise) in the Cox models, the time of diagnosis can be accounted for and immortal time bias can be eliminated^31^. In these Cox models, ICU discharge was treated as a censoring event, such that the exponentiated coefficient estimates could be interpreted as cause-specific hazard ratios. Unadjusted hazard ratios were estimated using Cox models which included a single time-varying indicator for whether a specific criterion had been reached. In addition, to assess the independent contribution of the SCrea and UO criteria, adjusted Cox models were fitted including separate time-varying indicators for each of these criteria. Finally, to assess the conditional association between diagnosis by each of the criteria and ICU mortality, accounting for other prognostic risk factors, a series of Cox models were fitted adjusted for gender, age and SOFA score on admission. The concordance index was reported for each fitted Cox model to enable a crude comparison in terms of (added) prognostic value of each of the (combined) criteria^32^. Additional details on statistical methods can be found in Supplementary Material S1. All analyses were conducted in R (version 3.6.0).

Our reporting of this study followed the STrengthening the Reporting of OBservational studies in Epidemiology (STROBE) reporting guidelines (supplementary material).

### Ethics approval and consent to participate

The Ghent University Hospital Ethics Committee approved the study (EC nr 201-0705) and waived informed consent since all analyses were performed retrospectively on pseudonymized records.

### Consent for publication

Not applicable.

## Results

We included 13,403 admissions, with demographic and baseline characteristics presented in Table 1. Of note, 84% of the cohort had an indwelling bladder catheter.

**Table 1:** Baseline demographic and clinical data.

Age (mean ± SD)	60.8 ± 16.8
Gender (% male)	62.2
Admission SOFA score (mean ± SD)	7.01 ± 4.1
Weight, kg (mean ± SD)	76.0 ± 16.7
Median ICU length of stay (hours) (Q1, Q3)	44 (23, 93)
Baseline SCrea (mean ± SD)	1.28 ± 2.7
Urinary catheter (%)	84.2
Chronic kidney disease (%)	16.3
Diabetes (%)	16.2

Depending on the applied criterion (or combination of criteria), incidence of AKI stage ≥ 2 diagnosis varied between 13.2 and 38.7% (Table 2). In Supplementary Table 2, we also list incidences based on the SCrea criterion depending on different choices of baseline SCrea measures, which ranged from 9.5% to 16.5%. Regardless of the applied criterion, patients with AKI vs no AKI were, on average, older, more likely to be male and to have diabetes or chronic kidney disease, and had a higher average SOFA score and weight at admission (Table 2). Of note, patients who were labelled as AKI based on the Screa criterium were more likely to have underlying CKD (40%) vs those labelled as AKI based on UO (20.3–27.0%).

**Table 2:** Incidence and demographic data according to different interpretations of the criteria for KDIGO AKI stage ≥ 2.

Criterion	Incidence of AKI (%)	Age (mean ± SD)		Admission SOFA score (mean ± SD)		Weight, kg (mean ± SD)		Baseline SCrea value, mg/dl (mean ± SD)		Urinary catheter (%)		Gender (% male)		Chronic kidney disease (%)		Diabetes (%)	
		AKI	No AKI	AKI	No AKI	AKI	No AKI	AKI	No AKI	AKI	No AKI	AKI	No AKI	AKI	No AKI	AKI	No AKI
SCrea	13.2	62.3 ± 15.1	60.6 ± 17.0	9.2 ± 4.5	6.7 ± 4.0	79.0 ± 17.6	75.6 ± 16.5	2.73 ± 3.12	1.05 ± 2.59			66.0	61.6	43.9	12.0	22.0	15.4
UO-1	34.3	64.1 ± 15.4	59.1 ± 17.2	8.6 ± 4.3	6.2 ± 3.8	81.4 ± 17.8	73.2 ± 15.3			86.2	81.4	66.6	59.9	20.3	14.1	20.1	14.2
UO-2	14.2	63.6 ± 15.6	60.3 ± 16.9	9.0 ± 4.6	6.7 ± 4.0	82.7 ± 19.3	74.9 ± 15.9			83.4	83.8	65.1	61.8	27.5	14.4	20.7	15.5
SCrea-UO-1	38.7	63.3 ± 15.6	59.2 ± 17.3	8.4 ± 4.3	6.2 ± 3.8	80.7 ± 17.7	73.1 ± 15.2					66.4	59.6	25.3	10.6	19.9	13.9
SCrea-UO-2	20.9	62.4 ± 15.7	60.4 ± 17.0	8.6 ± 4.5	6.6 ± 3.9	80.7 ± 18.6	74.8 ± 15.9					65.2	61.4	35.1	11.3	20.3	15.2

SCrea: serum creatinine > 4.0 mg/dl or > 2 × baseline, where baseline = SCrea-1 whenever available, otherwise SCrea-2, or SCrea-3 (when neither SCrea-1 nor SCrea-2 are available) with Screa-1 defined as baseline Screa measurement as manually entered in ICIS by the treating physician at ICU admission; Screa-2 defined as the lowest pre-ICU measurement up to 365 days before ICU admission as extracted from the lab information system; Screa-3 defined as a back-calculated baseline Screa using the simplified 4-variable Modification of Diet in Renal Disease (MDRD) Study equation assuming an estimated glomerular filtration rate (eGFR) of 75 ml/min/1.73 m^2^ for every patient^30^; UO-1: total UO during the last 12-h period was ≤ 6 ml/kg; UO-2: total UO during each of the last 12 consecutive 1-h periods was ≤ 0.5 ml/kg; SCrea-UO-1: AKI stage ≥ 2 according to either the SCrea criterion or the UO-1 criterion; SCrea-UO-2: AKI stage ≥ 2 according to either the SCrea criterion or the UO-2 criterion.

**Figure 1:**
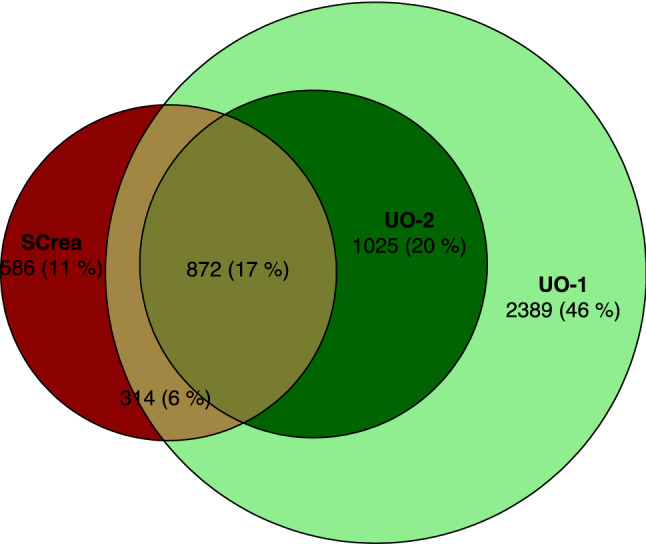
Euler diagram illustrating the distribution and overlap between AKI diagnosis according to different AKI criteria. Numbers indicate the number of patients in that overlap zone, so who would be diagnosed by different criteria. SCrea: serum creatinine > 4.0 mg/dl or > 2 × baseline, where baseline = SCrea-1 whenever available, otherwise SCrea-2, or SCrea-3 (when neither SCrea-1 nor SCrea-2 are available) with Screa-1 defined as baseline Screa measurement as manually entered in ICIS by the treating physician at ICU admission; Screa-2 defined as the lowest pre-ICU measurement up to 365 days before ICU admission as extracted from the lab information system; Screa-3 defined as a back-calculated baseline Screa using the simplified 4-variable Modification of Diet in Renal Disease (MDRD) Study equation assuming an estimated glomerular filtration rate (eGFR) of 75 ml/min/1.73 m^2^ for every patient^30^; UO-1: total UO during the last 12-h period was ≤ 6 ml/kg; UO-2: total UO during each of the last 12 consecutive 1-h periods was ≤ 0.5 ml/kg.

**Figure 2:**
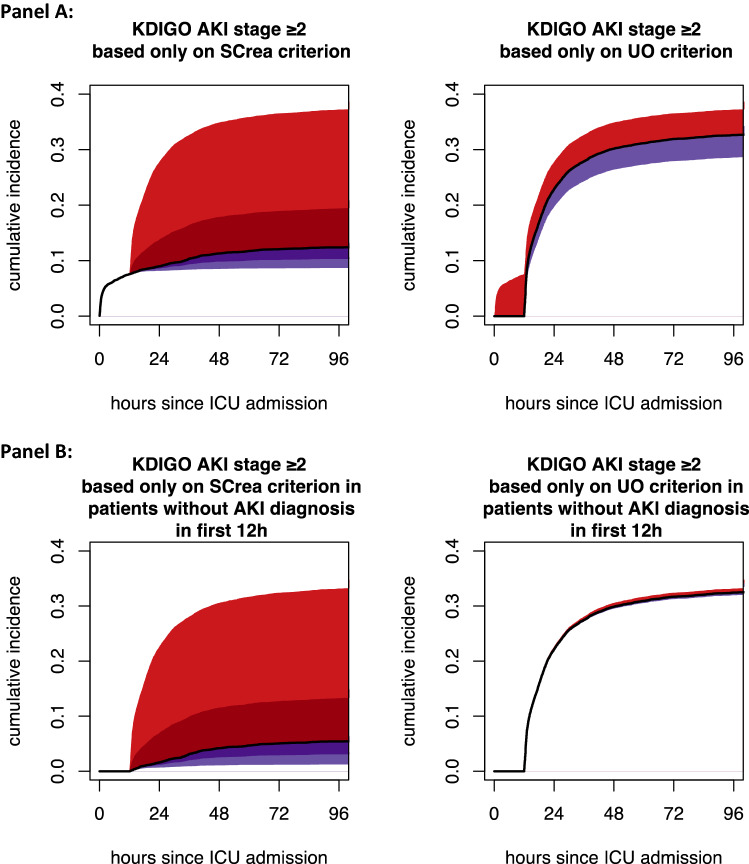
Cumulative incidence of automated AKI diagnosis over time. (**A**) Cumulative incidence of automated AKI diagnosis since ICU admission, based only on KDIGO Screa criterion (left) or only on KDIGO UO criterion (right) (black curves). Shaded areas represent cases that were, by each time point, either missed (red) or diagnosed with delay (purple) by ignoring the other criterion. (Dark shades in the left panel indicate missed or delayed cases compared to the UO-2 criterion, while the combination of light and dark shades in the left panel indicate missed or delayed cases compared to the UO-1 criterion). (**B**) Cumulative incidence of automated AKI diagnosis in patients still hospitalized and without AKI diagnosis by the 12th hour since ICU admission, based only on KDIGO Screa criterion (left) or only on KDIGO UO criterion (right) (black curves). Shaded areas represent cases that were, by each time point, either missed (red) or diagnosed with delay (purple) by ignoring the other criterion.

Figure 1 presents the distribution of AKI diagnoses according to the different criteria and their overlap. A minority of diagnoses (11%) is made only based on the SCrea criterion, while the majority of diagnoses (66%) is made only based on the UO criterion. This is also reflected by the red shaded areas in Fig. 2A, which represent the fraction of cases that would be missed over time by ignoring either the UO criterion (left panel) or the SCrea criterion (right panel). The black curves in Fig. 2A depict the cumulative incidence of AKI diagnosis based on the Screa (left panel) and the UO criterion (right panel) as a function of time since ICU admission (hours). As, by definition, patients cannot reach the UO criterion for AKI stage ≥ 2 before 12 h, initially diagnosis of AKI is made only based on the Screa criterion. The purple shaded areas in Fig. 2A represent the fraction of cases over time that are diagnosed with delay by ignoring either the UO criterion (left panel: about 12% of cases) or the SCrea criterion (right panel: about 11% of cases). Figure 2B displays the AKI incidence for patients not diagnosed with AKI within the first 12 h after ICU admission, which illustrates that later diagnosis (after 12 h) is almost exclusively made based on the UO criterion.

Table 3 displays ICU mortality incidence rates per 1,000 patient days at risk at the ICU with or without AKI diagnosis based on the different criteria (or combinations) and corresponding cause-specific hazard ratios (both unadjusted and adjusted for gender, age, and SOFA score at admission). Considering the UO criterion resulted in relatively higher relative hazards, as well as higher overall discriminative ability (as expressed by the concordance index), as compared to only considering the SCrea criterion. This pattern was found irrespective of whether the UO criterion was (1) strictly interpreted (UO-2) or more broadly (UO-1), (2) considered in combination with SCrea or not, or (3) adjusted for other risk factors or not.

**Table 3:** Incidence rates and hazard ratios of ICU mortality for AKI stage ≥ 2 vs no AKI stage ≥ 2 across different criteria.

KDIGO stage ≥ 2 criterion	Incidence rate of ICU mortality (estimated number of ICU deaths per 1,000 patient days at risk)^1^		Unadjusted cause-specific hazard ratio of ICU mortality^2^ (95% CI)	Concordance index^3^	Adjusted cause-specific hazard ratio of ICU mortality^4^ (95% CI)	Concordance index^3^
	Patient days with AKI	Patient days without AKI				
**Cox proportional hazards models including a single criterion as predictor**						
SCrea	31.3	16.1	2.11 (1.85–2.42)	0.568	1.81 (1.56–2.09)	0.663
UO-1	27.2	12.6	3.00 (2.55–3.54)	0.593	2.59 (2.18–3.09)	0.680
UO-2	36.6	14.1	3.21 (2.79–3.69)	0.597	2.83 (2.44–3.28)	0.688
SCrea-UO-1	26.5	12.1	2.85 (2.43–3.34)	0.604	2.54 (2.14–3.02)	0.683
SCrea-UO-2	32.8	13.2	2.93 (2.57–3.35)	0.615	2.62 (2.27–3.04)	0.695
**Cox proportional hazards model including both SCrea and UO-1 as predictors**						
SCrea			1.67 (1.46–1.92)	0.624	1.48 (1.27–1.71)	0.693
UO-1			2.63 (2.22–3.11)		2.35 (1.97–2.82)	
**Cox proportional hazards model including both SCrea and UO-2 as predictors**						
SCrea			1.35 (1.16–1.57)	0.624	1.18 (1.01–1.39)	0.694
UO-2			2.82 (2.41–3.29)		2.63 (2.23–3.10)	

^1^Incidence rate ratios only approximate (cause-specific) hazard ratios when the survival distributions in each group both follow an exponential distribution.

^2^Estimated by an extended Cox model that treats ICU discharge as a censoring event.

^3^Concordance indices are displayed for the corresponding Cox model whose exponentiated coefficient estimates are displayed in the column on the left hand side.

^4^Estimated by an extended Cox model that treats ICU discharge as a censoring event that additionally incorporates gender, age (binned into 4 categories according to quartiles) and SOFA score at ICU admission (binned into 4 categories according to quartiles). As a reference: the concordance index for a Cox model including only gender, age and SOFA score at ICU admission (excl. AKI criteria) equalled 0.640.

SCrea: serum creatinine > 4.0 mg/dl or > 2 × baseline, where baseline = SCrea-1 whenever available, otherwise SCrea-2, or SCrea-3 (when neither SCrea-1 nor SCrea-2 are available) with Screa-1 defined as baseline Screa measurement as manually entered in ICIS by the treating physician at ICU admission; Screa-2 defined as the lowest pre-ICU measurement up to 365 days before ICU admission as extracted from the lab information system; Screa-3 defined as a back-calculated baseline Screa using the simplified 4-variable Modification of Diet in Renal Disease (MDRD) Study equation assuming an estimated glomerular filtration rate (eGFR) of 75 ml/min/1.73 m^2^ for every patient^30^; UO-1: total UO during the last 12-h period was ≤ 6 ml/kg; UO-2: total UO during each of the last 12 consecutive 1-h periods was ≤ 0.5 ml/kg; SCrea-UO-1: AKI stage ≥ 2 according to either the SCrea criterion or the UO-1 criterion; SCrea-UO-2: AKI stage ≥ 2 according to either the SCrea criterion or the UO-2 criterion. An extended version of th table is included as Supplementary Table 2.

## Discussion

We observed in this study that incidence of AKI stage ≥ 2 diagnosis was higher and the association with ICU mortality stronger when the UO criterion is incorporated in the definition of AKI as compared to when it is not incorporated. We demonstrate the additive and independent predictive value of the UO to the Screa criterion with respect to ICU mortality. Accordingly, neglecting the UO criterion will result in underdiagnosis of AKI, may delay diagnosis of AKI and will underestimate the true relative hazard of ICU mortality associated with the label AKI.

To the best of our knowledge, this study is the first to take into account the time dynamics of AKI diagnosis by either algorithmic application of the SCrea criterion, the UO criterion, or both. Our approach of comparing cumulative incidence curves enabled to shed light on these dynamics and, in particular, to map, at each point in time, the number of potentially missed cases or delayed diagnoses by ignoring either the SCrea or the UO criterion. In particular, due to the time-dependent nature of the UO criterion, AKI presenting before or shortly after ICU admission can only be diagnosed using the SCrea criterion during the first 12 h. After 12 h, we observe a sudden steep increase in number of incident AKI diagnoses based almost exclusively on the UO criterion. Most of these new diagnoses based on the UO criterion would be missed by the SCrea criterion, whereas a small number would still be diagnosed by the SCrea criterion but only later on (Fig. 2B). This emphasizes the importance of the UO criterion for AKI detection after 12 h of admission, and by consequence, for the potential of (early) intervention. In sum, our results demonstrate that ignoring the UO criterion leads to a high number of missed casesand more delayed diagnoses in patients who did not yet have AKI at admission.

Comparisons of estimated associations between diagnosis by each of the criteria (or their combination) and ICU mortality demonstrate that AKI stage ≥ 2 diagnosis based on both the SCrea and UO criteria is more strongly associated with ICU mortality and has higher prognostic discriminative ability than diagnosis based on the SCrea criterion alone. This holds even after adjustment for other prognostic factors such as gender, age, and disease severity at ICU admission. These findings add emphasis on the importance of UO based on earlier reports on the association of UO alone with mortality^14,33^.

We used two different interpretations of the KDIGO urinary output criterion in our dataset: one more strict (oliguria as determined in each of 12 consecutive 1 h blocks) than the other (oliguria as determined in a moving total 12 h time window). Both options have previously been associated with mortality^14^. None of the patients who fulfilled the more strict criteria failed the broad criterion.

The different scenarios for baseline SCrea retrieval in our algorithms resulted in wide variation in AKI incidence (see Supplementary Table 1), confirming earlier findings^17^. Back-calculated values assuming a fixed eGFR, e.g. 75 ml/min/1,73m^2^, are often used^7,34–36^, but this approach leads to overestimation of AKI incidence, especially in the elderly and those with pre-existing CKD^37–41^. As we linked our ICU data system with the lab data system, we were able to retrieve SCrea values before index ICU hospitalization, a more valid baseline, in more than 80% of ICU admissions, which is much higher than reported in most other studies. However, patients suffering from chronic comorbidities and intermittent acute illnesses are more likely to have historical values available than healthy individuals. This is of importance as AKI algorithms will be most useful in cases where AKI is missed because the treating physician did not consider the diagnosis of AKI. A prediction tool that relies on whether a physician ordered Screa, and had thus a suspicion for AKI, will perform poorly for this purpose^42^. In addition, availability of measures may depend on local standards of care, which may contribute to poor external validity of the AKI algorithm^43^. The poor external validity of AKI detection algorithms has recently also been demonstrated in other series, where AKI was however only based on Screa change according to a baseline^8^.

Our findings have important implications for the interpretation of randomized as well as observational clinical studies or automated alert or prediction systems that use a definition of AKI that ignores UO. The use of such a truncated definition stands in sharp contrast with clinical reality, where many clinicians will diagnose AKI based on both Screa and UO. Our findings indicate that these missed cases based on omitting UO are clinically significant, which might be important in randomised controlled trials as opportunities for intervention might be missed. The last decade has witnessed an increased interest in automated alerting and decision support, sparked by the hope that such systems may lead to earlier diagnosis and better tailored management of patients with AKI. Different algorithms for prediction and/or automated alerting for AKI are already being developed^44^. Given that systematic reviews have highlighted the lack of uniformity and transparency in how AKI is defined within these models^7,9,11–13,45,46^, most of which ignore UO, it is important for clinicians to be aware of the implications of the use of such a truncated definition of AKI^26,27,33,47,48^. Current automated decision support systems for AKI may mislead clinicians, as they can no longer be confident on the meaning and implications of the diagnostic label. Our findings indicate that such automated systems should incorporate or be trained on databases that include the UO criterion in their AKI label, and that, accordingly, measurement and registration of UO in electronic health records could be an efficient way to earlier detect patients with or at risk of AKI, and to potentially improve outcomes. Devices that automatically monitor UO might be a worthwhile asset to improve the dismal outcome of AKI in ICU.

Our study is based on highly granular routinely collected data from a large patient cohort, extracted from an operational ICIS database of a large tertiary care ICU. Because this is a single center study however, it is still unclear whether current findings can be extrapolated or generalized to other settings.

From a theoretical perspective, UO could be misleading in patients with either bladder retention or urinary incontinence. However, in our cohort, nearly all patients (84%) had an indwelling bladder catheter, and there was no difference between those with vs without AKI. In anuric patients, a bladderscan is routinely performed to asses eventual retention. Also, it is standard practice in our unit to provide for hygienic reasons to provide an indwelling bladder catheter in patients with urinary incontinence.

A further strength of our analysis is that by accounting for the timing of AKI diagnosis, our analytic approach eliminated immortal time bias. This type of bias may have invalidated the results of other studies that assessed the association between different AKI criteria and adverse outcomes^15^.

For simplicity and ease of exposition, we have limited the presentation of our results to a single operational definition of the SCrea criterion that uses a specific order of potential choices of serum creatinine baseline based on their availability, as intended by the spirit of KDIGO-AKI definition. In Supplementary Tables 1 and 2 and Supplementary Fig. 1, we present results using a range of different choices of baseline. Many other alternative choices could however potentially have been investigated, such as the median of all pre-ICU creatinine values, or most recent Screa before hospital admission. We can thus not exclude that these other choices would lead to different results, although, given the wide range of considered baselines, this may be considered unlikely.

RRT initiation was not incorporated either in the SCrea criterion, nor included as endpoint because this was considered a rather subjective criterion and its incorporation would, moreover, have conflated the condition with its treatment. Moreover, based on daily practice in our unit, RRT would be started without AKI only in a few exceptional cases, such as lactic acidosis or intoxications irrespective of kidney function.

In conclusion, ignoring UO in the diagnosis of KDIGO AKI stage ≥ 2 decreases sensitivity, may lead to delayed diagnosis and results in underestimation of KDIGO AKI stage ≥ 2 associated mortality.

## Supplementary information

Supplementary Information 1.

Supplementary Information 2.

Supplementary Information 3.

Supplementary Information 4.

Supplementary Information 5.

Supplementary Information 6.

## Data Availability

The datasets generated and/or analysed during the current study are not publicly available due to potential privacy concerns but are available from the corresponding author (in pseudonymized format) on reasonable request.

## Abbreviations

AKI: Acute kidney injury
CKD: Chronic kidney disease
EHR: Electronic health records
ICIS: Intensive care information system
ICU: Intensive care unit
KDIGO: Kidney disease improving global outcomes
MDRD: Modification of diet in renal disease
SCrea: Serum creatinine
SOFA: Sequential organ failure assessment
UO: Urinary output
