# Supplementary material for: The importance of the urinary output criterion for the detection and prognostic meaning of AKI
Source: Sci Rep. 2021 May 27;11:11089. doi: 10.1038/s41598-021-90646-0 (PMC8159993; doi:10.1038/s41598-021-90646-0)
Supplement: Supplementary file 1 — Supplementary Information 1. [file 41598_2021_90646_MOESM1_ESM.docx]

**Supplementary Material S1**

***Patient population and data extraction***

All 52 beds of the Gent University Hospital (tertiary care) adult intensive care units (ICU) dispose of a commercially available Intensive Care Information System (ICIS) (Centricity Critical Care, GE Healthcare, Germany) to collect granular longitudinal patient data in real time. All data from monitors, ventilators, pumps, radiology results and laboratory results and administered medication are automatically uploaded in the system. In addition, a wide variety of pre-defined items are routinely entered manually by medical staff.

Raw data relevant for our study were extracted in a pseudonymized way from the ICIS database for all first ICU admissions between January 1, 2013 and December 31, 2017. Daily serum creatinine (mg/dl) (SCrea) and urinary output (UO) measurements during ICU stay were extracted from the ICIS, along with their corresponding sample times. For SCrea, this information originates from the lab information system directly linked to the ICIS. During ICU stay, measurements were taken at least daily, although some patients who died or were discharged from the ICU within 24 hours from admission may not have had any registered SCrea measurement after the time of ICU admission. Almost half of the patients (47.9%) had at least one ICU day of more intensive monitoring (i.e. >1 SCrea measurement). The study data set was complemented with all available SCrea values available to the lab information system up to 365 days before ICU admission. UO (ml) was periodically measured and entered into the ICIS by nursing staff who recorded the reading, mostly from a drainage container of a urinary catheter or from spontaneous urination in a scaled recipient (at fixed time intervals, or whenever the container was nearly full, at which point a valve was opened such that the urine was released into a larger container). Intensity of UO monitoring of each patient was decided by the treating physician and could be modified during ICU stay. 0.9% of all patients and 0.2% of patients admitted ≥ 24 hours did not have any UO measurements. 50% of recorded intervals between consecutive UO sample times were 1 hour or shorter, while 85% were 2 hours or shorter.

In addition, for each included patient, time from ICU admission to discharge, vital status at ICU discharge, gender, age on admission, weight, comorbidities (diabetes and chronic kidney disease), and Sequential Organ Failure Assessment (SOFA) score on the day of ICU admission were extracted.

SOFA scores were calculated as the sum of six SOFA subscores, each of which are scored from 0 to 4 and are calculated in real-time in the ICIS for each 24h interval from 6am of the current calendar day to 6am the next calendar day, based on available lab results imported in the ICIS, physiological parameters and administered drugs and interventions (as detailed in [1]). The six SOFA subscores reflect

- coagulation function
- renal function
- cardiovascular system function
- central nervous system function
- hepatic system function
- respiratory function

Higher scores reflect increasing levels of organ dysfunction. However, this score may in part reflect the subjective appraisal of a patient’s condition by intensive care physicians because when a patient is not suspected to have a particular organ dysfuntion no tests are ordered beyond routine test and measurement procedures. Corresponding subscores that therefore may be missing are scored 0.

***Ethical approval***

Ethical approval was obtained from the Ethical Committee of Ghent University Hospital (EC nr 201-0705).

***Primary endpoints***

The KDIGO criteria for AKI stage ≥ 2 diagnosis hinge on two different parameters: 1/ absolute or relative increase in SCrea as compared to a baseline measurement (Screa criterion) and 2/ oliguria during a 12 hour period (UO criterion). As primary endpoints we considered (cumulative) incidence of AKI stage ≥ 2 diagnosis during ICU stay as defined by the KDIGO guidelines, based on either criterion, or both, and the association between diagnosis by each of these criteria and ICU mortality, as a crude reflection of prognostic value. Even though classification of AKI diagnosis according to the different criteria was done retrospectively on electronic health records, the automated computer code that was used could in principle also be prospectively be applied to detect or alert AKI in real-time if incorporated in an intensive care information system.

***Data preprocessing: automated retrospective diagnosis of AKI stage≥2***

For each patient we assessed whether and when any SCrea measurement was found during their ICU stay that either exceeded 4.0 mg/dl or indicated a >2-fold increase relative to one of the following baseline Screa measurements, selected in the order listed below, according to availability:

- **SCrea-1** A baseline measurement as manually entered in the ICIS by the treating physician at ICU admission. Physicians are explicitly asked to *always* enter a (documented) SCrea measurement before the current hospitalization and no older than 365 days or, if not available, the lowest measurement of the current hospitalization. In other words: baseline measurements could be retrieved from lab results from previous hospitalizations (at Ghent University Hospital or at another center, e.g. via external sources), whichever was available to the treating physician*.* In case multiple baseline values were entered, only the last was retained, under the assumption that more recent entries could be considered as corrections to previous entries. Because these levels were entered manually, a number of entries can be considered unreliable (0.2% of registered values exceeded the biologically plausible maximum of 15 mg/dl). This is suspected to be most often due to wrongly placed decimals (resulting in levels with a measurement error of a factor 10 or 100).
- **SCrea-2** The lowest (available) pre-ICU SCrea measurement up to 365 days before ICU admission as extracted from the lab information system. This could be a value from before or during the index hospitalisation but before admission to ICU.
- **SCrea-3** A back-calculated baseline SCrea using the simplified 4-variable Modification of Diet in Renal Disease (MDRD) Study equation assuming an estimated glomerular filtration rate (eGFR) of 75 ml/min/1.73 m^2^ for every patient [2]

$$SCrea= \left( \frac{\mathrm{eGFR}}{186 \times0.742 (if female) \times1.21 (if black) \times{age (years)}^{-0.203}} \right)^{-\frac{1}{1.154}}$$

As race was missing from the ICIS and only a small minority of ICU patients at Ghent University Hospital is black, every patient was assumed to be non-black. Any other formula, for example CKD-EPI, could have been used, as our aim was not to find the best way to define AKI, but rather to illustrate that the choice of baseline impacts incidence and prognostic value.

In the interest of space, the main text only provides results for automated AKI diagnosis based on implementation of the Screa criterion relative to a baseline selected by this stepwise approach (henceforth labelled **Screa**). In Supplementary Tables 1 and 2, we also present results for automated AKI diagnosis based on implementation of the Screa criterion relative to each of these three potential baseline measures separately, as well as results for diagnosis based on the SCrea criterion relative the following alternative baseline measures:

- **SCrea-4** The lowest (available) pre-ICU SCrea measurement of the current hospitalization as extracted from the lab information system.
- **SCrea-5** The SCrea level from the first blood sample taken since ICU admission as extracted from the lab information system.

In case a baseline SCrea value was missing, AKI could technically not be staged according to the corresponding operational definition. However, to mimic the logic of an automated detection system, AKI stage≥2 diagnosis with respect to that particular SCrea baseline was in that case coded as absent. Pre-ICU SCrea levels extracted from the lab information system were first converted into numerical values. In a number of cases, this was not possible, because entries included comments and free text. In case entries indicated that SCrea levels were below a certain detection threshold (e.g. <0.17 mg/dl or <0.20 mg/dl), SCrea levels were set to the corresponding detection threshold.

To retrospectively identify patients with AKI stage≥2 based on the UO criterion, UO measurements were first converted to ml/kg/h taking into account patients’ weight and the length of recorded UO intervals. Patients’ weight was entered into the ICIS by nursing staff based on recent weight registrations when available in the hospital information system, self-report from the patient, from relatives, or an estimated clinical guess. Two hundred forty eight (248) patients had a registered weight of 0 kg or less. Since their weight registration is considered unreliable, their weights were set to missing.

For each patient we assessed whether and when any UO registration was found during their ICU stay at which

- **UO-1** total UO during the last 12-hour period was ≤ 6 ml/kg
- **UO-2** total UO during each of the last 12 consecutive 1-hour periods was ≤ 0.5 ml/kg

Both operational definitions are compatible with the KDIGO guidelines for AKI stage≥2 diagnosis. Below we describe in more detail how UO was calculated in X-hour time windows.

Consider, for each patient, a series of UO samples, denoted *k*, ranging from 1 to *K*, with *K* the total number of samples for a single patient. The 12-hour period (UO-1) corresponding to each evaluated sample time *t_k_* was defined as the time window starting exactly 12 hours before that sample time, i.e. *t_k_-12h*, and ending at *t_k_*. This time window spanned the 12 consecutive 1-hour periods (UO-2), which were also each aligned with respect to the evaluated sample time (i.e. [*t_k_-12h, t_k_-11h*]*,* [*t_k_-11h, t_k_-10h*]*, …,* [*t_k_-1h, t_k_*]). Note that, because UO measurements are unavailable before ICU admission, diagnosis based on the UO criterion could only be empirically established as soon as patients had been hospitalized at the ICU for at least 12 hours. Moreover, because intervals defined by consecutive sample times *t_k_* rarely aligned with 1-hour time windows, UO within each considered time window was estimated by a linear interpolation approach.

We considered the minimal number *q* of previous sample times such that the consecutive UO sample intervals from *t_k-q_* to *t_k_* jointly exceed a total duration of 1 hour, i.e. duration of [*t_k-q_*, *t_k_*] > duration of [*t_k_-1h*, *t_k_*] = 1h, but duration of [*t_k-q+1_*, *t_k_*] < duration of [*t_k_-1h*, *t_k_*] = 1h. UO during the 1-hour period [*t_k_-1h*, *t_k_*] was then set to the total UO in the interval [*t_k-q_*, *t_k_*] subtracted by the linearly interpolated UO in the interval [*t_k-q_*, *t_k_-1h*], which corresponds to the UO rate (ml/kg/h) in the interval [*t_k-q_*, *t_k-q+1_*] multiplied by the duration of [*t_k-q_*, *t_k_-1h*] (expressed in hours). For instance, whenever the interval [*t_k-1_*, *t_k_*] exceeded 1 hour, *q* = 1, and the UO during the 1-hour period [*t_k_-1h*, *t_k_*] was simply set to the UO rate (ml/kg/h) of the interval [*t_k-1_*, *t_k_*]. Whenever the interval [*t_k-1_*, *t_k_*] did not exceed 1 hour, *q* > 1, and the UO during the 1-hour period [*t_k_-1h*, *t_k_*] was set to the total UO in the interval [*t_k-q_*, *t_k_*] subtracted by the linearly interpolated UO in the interval [*t_k-q_*, *t_k_-1h*] derived from the UO rate (ml/kg/h) in the interval [*t_k-q_*, *t_k-q+1_*]. A similar procedure was followed to retrospectively calculate UO (ml/kg) in each of the consecutive 1-h intervals [*t_k_-12h, t_k_-11h*]*,* [*t_k_-11h, t_k_-10h*]*, …,* [*t_k_-2h, t_k_-1h*]. For instance, to calculate UO in the interval [*t_k_-2h, t_k_-1h*], the aforementioned procedure was followed to first calculate UO in the interval [*t_k_-2h, t_k_*], with *q* now being the minimal number of sample times before *k* such that the consecutive UO sample intervals from *t_k-q_* to *t_k_* jointly exceed a total duration of 2 hours, from which calculated UO in the interval [*t_k_-1h, t_k_*] was subtracted. The same recursive logic was applied to calculate UO in the consecutive 1-h intervals [*t_k_-12h, t_k_-11h*]*,* [*t_k_-11h, t_k_-10h*]*, …,* [*t_k_-3h, t_k_-2h*]. Finally, to calculate UO in the interval [*t_k_-12h, t_k_*] the same procedure was followed, with *q* being the minimal number of sample times before *k* such that the consecutive UO sample intervals from *t_k-q_* to *t_k_* jointly exceed a total duration of 12 hours.

In addition, to assess the relative impact of ignoring either of these criteria, two composite definitions of AKI stage≥2 were postulated:

- **Screa-UO-1**, which indicates whether a patient is diagnosed based on either the Screa criterion or the UO-1 criterion (as defined above)
- **Screa-UO-2**, which indicates whether a patient is diagnosed based on either the Screa criterion or the UO-2 criterion (as defined above).

In a limited number of patients, no SCrea (n=231; 1.7%) or no UO (n=122; 0.9%) measurements were recorded. It was assumed that either length of stay was too short for any measurements to be available during ICU stay (in case of SCrea) or that the treating physician deemed regular UO monitoring and registration in these patients unnecessary. AKI diagnosis based on any of the above criteria or its different interpretations was therefore assumed absent in these patients (unless it could be based on the complementary criterion, for the composite definitions SCrea-UO-1 and SCrea-UO-2). In sum, AKI diagnosis was only coded as present according to a specific criterion if that criterion was clearly met, and coded as absent whenever that criterion was either not met or insufficient data were available to evaluate whether the criterion was met. No patients or episodes were excluded for analysis based on whether any data for criterion evaluation was missing. In Supplementary Table 4, we give an overview of the percentage of missing data for evaluation for each of the criteria and its impact on incidence of AKI.

***Cumulative incidence curves for AKI stage≥2 as diagnosed by different criteria and calculation of percentage of missed cases or delayed diagnoses***

Incidences of AKI stage≥2 as diagnosed by each separate criterion were compared. To more closely inspect dynamics over time, cumulative incidence functions, as estimated by the Aalen-Johansen estimator (treating ICU death and discharge as competing events), were obtained and compared. Comparison of these curves enabled to retrospectively assess, at each time point, the percentage of cases (that would be diagnosed by using both criteria) that were either missed or diagnosed with delay by ignoring either the SCrea or the UO criterion. More specifically, we obtained cumulative incidence curves for automated AKI stage ≥2 diagnosis based on

- either the SCrea or UO criterion, whichever occurred first (A);
- only the SCrea criterion, ignoring diagnosis by the UO criterion (B);
- only the UO criterion, ignoring diagnosis by the SCrea criterion (C);
- only the SCrea criterion, treating earlier diagnosis by the UO criterion as a competing event (D);
- only the UO criterion, treating earlier diagnosis by the SCrea criterion as a competing event (E).

Curves D and E express the cumulative incidences of AKI stage≥2 as diagnosed *first* based on a particular criterion, while curves B and C ignore the potential order of time points at which separate criteria for diagnosis are met (in case both criteria are met before ICU death or discharge). The contrast of curves A and B captures, by each time point, the percentage of missed cases in the entire cohort (= #missed cases/n, where n = size of the cohort) when ignoring the UO criterion (as compared to considering both criteria for automated diagnosis). The contrast of curves A and C captures, by each time point, the percentage of missed cases in the entire cohort when ignoring the SCrea criterion (as compared to considering both criteria for automated diagnosis). The contrast of curves B and D captures, by each time point, the percentage of cases that were detected by the SCrea criterion, but that were detected earlier by the UO criterion (i.e. delayed diagnosis as compared to the UO criterion). The contrast of curves C and E captures, by each time point, the percentage of cases that were detected by the UO criterion, but that were detected earlier by the SCrea criterion (i.e. delayed diagnosis as compared to the SCrea criterion). Curves A, C and E were obtained both with respect to the broad interpretation (UO-1) and the strict interpretation of the UO criterion (UO-2) to assess the number of missed cases or delayed diagnoses based only on the SCrea criterion when ignoring either UO-1 or UO-2. Conversely, the number of reported missed cases or delayed diagnoses based only on the UO criterion when ignoring the SCrea criterion was calculated only with respect to the broad interpretation of the UO criterion (UO-1).

Because no urinary output measurements were available before ICU admission, AKI diagnosis based only on the UO criterion could occur no sooner than 12 hours after ICU admission. Because this ‘artefact' gives a 'head start' to the SCrea criterion as only potential criterion for automated AKI stage≥2 diagnosis within the first 12 hours of ICU admission, the above comparisons were also made in patients still hospitalized and without AKI diagnosis by the 12th hour since ICU admission. Results in this subcohort enable a comparison between the criteria that is adjusted for the fact that the UO criteria may (otherwise) in some patients lead to a delayed diagnosis as compared to the SCrea criterion purely due to the aforementioned artefact.

***Association with ICU mortality and discriminative ability of different diagnostic criteria for KDIGO AKI stage≥2***

The associations between diagnosis by each of the aforementioned criteria and ICU mortality, as captured by cause-specific hazard ratios comparing patient time at risk of ICU mortality with versus without diagnosis, were estimated using a series of extended Cox proportional hazards models for time from admission to ICU death. Other regression methods, such as logistic regression, cannot account for the timing of AKI diagnosis and therefore artificially prevent AKI diagnosed patients from dying before their diagnosis (i.e. during a so-called 'immortal time period’ from admission to AKI diagnosis). The artificially elevated survival rate in AKI patients results in immortal time bias, which may erroneously underestimate the risk (or odds) ratio associated with AKI diagnosis and lead to incorrect inferences. This is particularly problematic for the UO criterion because, as a consequence of the time-dependent nature of this criterion (which requires an oliguric period of at least 12 hours), patients with diagnosis based on the UO criteria by definition survived the first 12 hours of their ICU stay. By including AKI stage≥2 diagnosis as a time-varying rather than a time-fixed covariate (coded 1 from the time of AKI diagnosis and 0 otherwise) in the Cox models, the time of diagnosis can be accounted for and immortal time bias can be eliminated [3]. In these Cox models, ICU discharge was treated as a censoring event, such that the exponentiated coefficient estimates could be interpreted as cause-specific hazard ratios. ICU discharge was treated as a censoring rather than a competing event because the latter approach fails to adjust for the fact that potentially prolonged length of stay at the ICU indirectly increases the risk of eventually dying at the ICU. In Supplementary Table 2, we also report subdistribution hazard ratios, as estimated by Fine-Gray Cox models that treat ICU discharge as a competing event. These hazard ratios also capture the indirect association between AKI diagnosis and ICU mortality through potentially prolonged ICU stay. For both approaches, corresponding incidence rates and incidence rate ratios are reported. When treating ICU discharge as a censoring event, the denominator of the incidence rate corresponds to patient days at risk during ICU stay. When treating ICU discharge as a competing event, this corresponds to patient days at risk (including days after ICU discharge).

Unadjusted hazard ratios were estimated using Cox models which included a single time-varying indicator for whether a specific criterion had been reached. In addition, to assess the independent contribution of the SCrea and UO criteria, adjusted Cox models were fitted including separate time-varying indicators for each of these criteria. Finally, to assess the conditional association between diagnosis by each of the criteria and ICU mortality, accounting for other prognostic risk factors, a series of Cox models were fitted adjusted for gender, age and SOFA score on admission. The concordance index, which reflects the discriminative ability of a prognostic marker (or a combination of markers), was reported for each fitted Cox model to enable a crude comparison in terms of (added) prognostic value of each of the (combined) criteria. This rank-based measure can be considered an extension of the AUC for longitudinal data. As it has been shown to be a weighted average of time-dependent AUCs [4], it can be interpreted in a similar fashion as the AUC (with a value of 0.5 indicating no discriminative ability and a value of 1 indicating perfect discriminative ability).

***Cautionary notes on interpretation of hazard ratios and concordance indices***

Despite the fact that our analytical approach for estimating the association between diagnosis by different AKI criteria and ICU mortality eliminates immortal time bias, results should be interpreted with caution. In particular, it has been pointed out that the magnitude of hazard ratios provide a generally poor reflection of prognostic value (e.g. [5]). To accommodate this concern, we additionally report concordance indices. This metric provides a better yet (still) partial reflection of prognostic value as it only reflects discrimination but not calibration (e.g. [6]). Moreover, strength of association or discriminative ability may depend on both the time at which the criterion was first met and the duration for which the criterion is met. Reported hazard ratios and concordance indices only provide a weighted average of their time-varying counterparts. As such, these measures do not enable to distinguish which criterion has higher prognostic value at which point in time and may therefore provide an oversimplified summary of clinical reality. While our study provides a detailed mapping of the time dynamics of incidence of AKI diagnosis by different criteria, future studies using appropriate methodology may aid to further extend this mapping to the time dynamics of the prognostic value of these criteria [7,8].

**References**

1. Houthooft R, Ruyssinck J, van der Herten J, Stijven S, Couckuyt I, Gadeyne B, et al. Predictive modelling of survival and length of stay in critically ill patients using sequential organ failure scores. Artif Intell Med [Internet]. Elsevier B.V.; 2015;63:191–207. Available from: http://dx.doi.org/10.1016/j.artmed.2014.12.009

2. Levey AS, Greene T, Kusek J, Beck G. A simplified equation to predict glomerular filtration rate from serum creatinine [Abstract]. J Am Soc Nephrol. 2000;11:155A.

3. Shintani AK, Girard TD, Eden SK, Arbogast PG, Moons KGM, Ely EW. Immortal time bias in critical care research: Application of time-varying Cox regression for observational cohort studies*. Crit Care Med [Internet]. 2009;37:2939–45. Available from: http://content.wkhealth.com/linkback/openurl?sid=WKPTLP:landingpage&an=00003246-200911000-00011

4. Heagerty PJ, Zheng Y. Survival model predictive accuracy and ROC curves. Biometrics. 2005;61:92–105.

5. Vickers AJ, Cronin AM. Everything you always wanted to know about evaluating prediction models (but were too afraid to ask). Urology [Internet]. Elsevier Inc.; 2010;76:1298–301. Available from: http://dx.doi.org/10.1016/j.urology.2010.06.019

6. Kattan MW, Gerds TA. The index of prediction accuracy: an intuitive measure useful for evaluating risk prediction models. Diagnostic Progn Res. Diagnostic and Prognostic Research; 2018;2:1–7.

7. Bansal A, Heagerty PJ. A Tutorial on Evaluating the Time-Varying Discrimination Accuracy of Survival Models Used in Dynamic Decision Making. Med Decis Mak. 2018;38:904–16.

8. Bansal A, Heagerty PJ. A comparison of landmark methods and time-dependent ROC methods to evaluate the time-varying performance of prognostic markers for survival outcomes. Diagnostic Progn Res. Diagnostic and Prognostic Research; 2019;3.
