# Supplementary material for: The importance of the urinary output criterion for the detection and prognostic meaning of AKI
Source: Sci Rep. 2021 May 27;11:11089. doi: 10.1038/s41598-021-90646-0 (PMC8159993; doi:10.1038/s41598-021-90646-0)
Supplement: Supplementary file 2 — Supplementary Information 2. [file 41598_2021_90646_MOESM2_ESM.docx]

**Supplementary Table 1: Incidence and demographic data according to different criteria for KDIGO AKI stage ≥2 (including different choices of SCrea baseline)**

[Trek de aandacht van uw lezer met een veelzeggend citaat uit het document of gebruik deze ruimte om een belangrijk punt te benadrukken. Sleep dit tekstvak als u het ergens anders op de pagina wilt plaatsen.]

|  | **Incidence of AKI (%)** | **Age (mean±SD)** | | **Admission SOFA score (mean±SD)** | | **Weight, kg (mean±SD)** | | **Baseline SCrea value, mg/dl (mean±SD)** | | **Gender  (% male)** | | **Chronic  kidney disease (%)** | | **Diabetes (%)** | |
| --- | --- | --- | --- | --- | --- | --- | --- | --- | --- | --- | --- | --- | --- | --- | --- |
|  |  | **AKI** | **No AKI** | **AKI** | **No AKI** | **AKI** | **No AKI** | **AKI** | **No AKI** | **AKI** | **No AKI** | **AKI** | **No AKI** | **AKI** | **No AKI** |
| *SCrea-1* | 11.4 | 62.0±15.0 | 60.6±17.0 | 9.0±4.4 | 6.8±4.0 | 79.1±17.0 | 75.6±16.6 | 2.87±3.11 | 1.07±2.88 | 68.0 | 61.5 | 48.2 | 12.1 | 22.7 | 15.4 |
| *SCrea-2* | 13.0 | 62.2±15.3 | 60.6±16.9 | 8.9±4.4 | 6.7±4.0 | 78.1±17.3 | 75.7±16.5 | 2.67±3.10 | 0.94±0.47 | 65.9 | 61.7 | 47.3 | 11.6 | 22.2 | 15.3 |
| *SCrea-3* | 16.5 | 65.0±14.8 | 60.0±17.0 | 9.0±4.4 | 6.6±4.0 | 78.5±17.3 | 75.5±16.5 | 0.97±0.13 | 0.99±0.13 | 62.8 | 62.1 | 54.7 | 8.6 | 25.2 | 14.5 |
| *SCrea-4* | 10.8 | 62.3±15.2 | 60.6±16.9 | 9.0±4.4 | 6.8±4.0 | 78.7±16.8 | 75.7±16.6 | 3.54±3.59 | 1.03±0.57 | 67.3 | 61.6 | 50.7 | 12.1 | 22.3 | 15.5 |
| *SCrea-5* | 9.5 | 61.9±15.3 | 60.7±16.9 | 8.8±4.4 | 6.8±4.1 | 79.1±16.4 | 75.7±16.7 | 4.00±3.15 | 0.99±0.52 | 68.6 | 61.5 | 53.4 | 12.3 | 21.9 | 15.6 |
| SCrea | 13.2 | 62.3±15.1 | 60.6±17.0 | 9.2±4.5 | 6.7±4.0 | 79.0±17.6 | 75.6±16.5 | 2.73±3.12 | 1.05±2.59 | 66.0 | 61.6 | 43.9 | 12.0 | 22.0 | 15.4 |
| UO-1 | 34.3 | 64.1±15.4 | 59.1±17.2 | 8.6±4.3 | 6.2±3.8 | 81.4±17.8 | 73.2±15.3 |  |  | 66.6 | 59.9 | 20.3 | 14.1 | 20.1 | 14.2 |
| UO-2 | 14.2 | 63.6±15.6 | 60.3±16.9 | 9.0±4.6 | 6.7±4.0 | 82.7±19.3 | 74.9±15.9 |  |  | 65.1 | 61.8 | 27.5 | 14.4 | 20.7 | 15.5 |
| SCrea-UO-1 | 38.7 | 63.3±15.6 | 59.2±17.3 | 8.4±4.3 | 6.2±3.8 | 80.7±17.7 | 73.1±15.2 |  |  | 66.4 | 59.6 | 25.3 | 10.6 | 19.9 | 13.9 |
| SCrea-UO-2 | 20.9 | 62.4±15.7 | 60.4±17.0 | 8.6±4.5 | 6.6±3.9 | 80.7±18.6 | 74.8±15.9 |  |  | 65.2 | 61.4 | 35.1 | 11.3 | 20.3 | 15.2 |

***Screa-1*** *SCrea >4.0 mg/dl or >2x baseline as manually entered in ICIS by the treating physician at ICU admission;* ***Screa-2*** *SCrea >4.0 mg/dl or >2x baseline defined as lowest pre-ICU measurement up to 365 days before ICU admission as extracted from the lab information system;* ***Screa-3*** *SCrea >4.0 mg/dl or >2x back-calculated baseline calculated using the simplified 4-variable Modification of Diet in Renal Disease (MDRD) Study equation assuming an estimated glomerular filtration rate (eGFR) of 75 ml/min/1.73 m^2^ for every patient;* ***Screa-4*** *SCrea >4.0 mg/dl or >2x baseline defined as lowest pre-ICU measurement of the current hospitalization as extracted from the lab information system****; Screa-5*** *SCrea >4.0 mg/dl or >2x baseline defined as the first measurement taken since ICU admission as extracted from the lab information system;* ***SCrea****: serum creatinine >4.0 mg/dl or >2x baseline, where baseline corresponds to that defined in SCrea-1 whenever available, otherwise SCrea-2, or SCrea-3 (when neither SCrea-1 nor SCrea-2 are available);* ***UO-1:*** *total* *UO during the last 12-hour period was ≤ 6 ml/kg;* ***UO-2:*** *total* *UO during each of the last 12 consecutive 1-hour periods was ≤ 0.5 ml/kg;* ***SCrea-UO-1****: AKI stage≥2 according to either the SCrea criterion or the UO-1 criterion;* ***SCrea-UO-2****: AKI stage≥2 according to either the SCrea criterion or the UO-2 criterion.*
