# Supplementary material for: The importance of the urinary output criterion for the detection and prognostic meaning of AKI
Source: Sci Rep. 2021 May 27;11:11089. doi: 10.1038/s41598-021-90646-0 (PMC8159993; doi:10.1038/s41598-021-90646-0)
Supplement: Supplementary file 3 — Supplementary Information 3. [file 41598_2021_90646_MOESM3_ESM.docx]

**Supplementary Table 2: Incidence rates and hazard ratios of ICU mortality for AKI stage≥2 vs no AKI stage≥2 (extended version of Table 3 in main text)**

[Trek de aandacht van uw lezer met een veelzeggend citaat uit het document of gebruik deze ruimte om een belangrijk punt te benadrukken. Sleep dit tekstvak als u het ergens anders op de pagina wilt plaatsen.]

| KDIGO stage≥2 criterion | Incidence rate of ICU mortality  (estimated number of ICU deaths per 1,000 patient days at risk) | | Incidence rate ratio of ICU mortality^1^  (95% CI) | Unadjusted cause-specific hazard ratio of ICU mortality^2^ (95% CI) | Concordance index^3^ | Adjusted  cause-specific hazard ratio of ICU mortality^4^ (95% CI) | Concordance index^3^ | Unadjusted cause-specific hazard ratio of ICU discharge^2^ (95% CI) | Unadjusted subdistribution hazard ratio of ICU mortality^5^ (95% CI) | Concordance index^3^ |
| --- | --- | --- | --- | --- | --- | --- | --- | --- | --- | --- |
|  | Patient days  with AKI | Patient days without AKI |  |  |  |  |  |  |  |  |
| *Cox proportional hazards models including a single criterion as predictor* | | | | | | | | | | |
| SCrea-1 | 29.5 | 17.3 | 1.71 (1.49-1.95) | 1.81 (1.57-2.08) | 0.545 | 1.55 (1.34-1.81) | 0.654 | 0.62 (0.58-0.66) | 4.13 (3.61-4.73) | 0.598 |
| SCrea-2 | 29.9 | 17.1 | 1.75 (1.54-2.00) | 1.82 (1.59-2.09) | 0.557 | 1.55 (1.33-1.79) | 0.657 | 0.65 (0.62-0.69) | 3.74 (3.28-4.28) | 0.599 |
| SCrea-3 | 31.2 | 15.4 | 2.02 (1.79-2.29) | 2.17 (1.91-2.47) | 0.581 | 1.77 (1.54-2.04) | 0.663 | 0.57 (0.55-0.61) | 4.72 (4.17-5.34) | 0.643 |
| SCrea-4 | 29.1 | 17.6 | 1.65 (1.44-1.90) | 1.72 (1.49-1.98) | 0.543 | 1.45 (1.24-1.70) | 0.651 | 0.66 (0.62-0.70) | 3.70 (3.22-4.26) | 0.584 |
| SCrea-5 | 26.3 | 18.4 | 1.43 (1.23-1.66) | 1.51 (1.29-1.76) | 0.515 | 1.35 (1.15-1.59) | 0.644 | 0.71 (0.66-0.75) | 3.51 (3.02-4.07) | 0.570 |
| SCrea | 31.3 | 16.1 | 1.94 (1.71-2.20) | 2.11 (1.85-2.42) | 0.568 | 1.81 (1.56-2.09) | 0.663 | 0.58 (0.54-0.61) | 5.11 (4.50-5.80) | 0.631 |
| UO-1 | 27.2 | 12.6 | 2.16 (1.90-2.46) | 3.00 (2.55-3.54) | 0.593 | 2.59 (2.18-3.09) | 0.680 | 0.61 (0.58-0.63) | 8.02 (6.88-9.36) | 0.704 |
| UO-2 | 36.6 | 14.1 | 2.59 (2.29-2.92) | 3.21 (2.79-3.69) | 0.597 | 2.83 (2.44-3.28) | 0.688 | 0.55 (0.52-0.58) | 9.52 (8.35-10.85) | 0.681 |
| SCrea-UO-1 | 26.5 | 12.1 | 2.19 (1.92-2.51) | 2.85 (2.43-3.34) | 0.604 | 2.54 (2.14-3.02) | 0.683 | 0.62 (0.60-0.65) | 6.53 (5.66-7.53) | 0.700 |
| SCrea-UO-2 | 32.8 | 13.2 | 2.48 (2.20-2.81) | 2.93 (2.57-3.35) | 0.615 | 2.62 (2.27-3.04) | 0.695 | 0.59 (0.56-0.62) | 6.88 (6.07-7.79) | 0.694 |
| *Cox proportional hazards model including both SCrea and UO-1 as predictors* | | | | | | | | | | |
| SCrea |  | | | 1.67 (1.46-1.92) | 0.624 | 1.48 (1.27-1.71) | 0.693 | 0.66 (0.62-0.70) | 3.14 (2.75-3.58) | 0.740 |
| UO-1 |  |  |  | 2.63 (2.22-3.11) |  | 2.35 (1.97-2.82) |  | 0.66 (0.63-0.68) | 6.26 (5.35-7.34) |  |
| *Cox proportional hazards model including both SCrea and UO-2 as predictors* | | | | | | | | | | |
| SCrea |  | | | 1.35 (1.16-1.57) | 0.624 | 1.18 (1.01-1.39) | 0.694 | 0.68 (0.64-0.72) | 2.35 (2.03-2.71) | 0.710 |
| UO-2 |  |  |  | 2.82 (2.41-3.29) |  | 2.63 (2.23-3.10) |  | 0.64 (0.60-0.68) | 6.72 (5.80-7.79) |  |

***Screa-1*** *SCrea >4.0 mg/dl or >2x baseline as manually entered in ICIS by the treating physician at ICU admission;* ***Screa-2*** *SCrea >4.0 mg/dl or >2x baseline defined as lowest pre-ICU measurement up to 365 days before ICU admission as extracted from the lab information system;* ***Screa-3*** *SCrea >4.0 mg/dl or >2x back-calculated baseline calculated using the simplified 4-variable Modification of Diet in Renal Disease (MDRD) Study equation assuming an estimated glomerular filtration rate (eGFR) of 75 ml/min/1.73 m^2^ for every patient;* ***Screa-4*** *SCrea >4.0 mg/dl or >2x baseline defined as lowest pre-ICU measurement of the current hospitalization as extracted from the lab information system****; Screa-5*** *SCrea >4.0 mg/dl or >2x baseline defined as the first measurement taken since ICU admission as extracted from the lab information system;* ***SCrea****: serum creatinine >4.0 mg/dl or >2x baseline, where baseline corresponds to that defined in SCrea-1 whenever available, otherwise SCrea-2, or SCrea-3 (when neither SCrea-1 nor SCrea-2 are available);* ***UO-1:*** *total* *UO during the last 12-hour period was ≤ 6 ml/kg;* ***UO-2:*** *total* *UO during each of the last 12 consecutive 1-hour periods was ≤ 0.5 ml/kg;* ***SCrea-UO-1****: AKI stage≥2 according to either the SCrea criterion or the UO-1 criterion;* ***SCrea-UO-2****: AKI stage≥2 according to either the SCrea criterion or the UO-2 criterion.*

^1^ Incidence rate ratios only approximate (cause-specific) hazard ratios when the survival distributions in each group both follow an exponential distribution.

^2^ Estimated by an extended Cox model (that treats ICU discharge as a censoring event)

^3^ Concordance indices are displayed for the corresponding Cox model whose exponentiated coefficient estimates are displayed in the column on the left hand side.

^4^ Estimated by an extended Cox model (that treats ICU discharge as a censoring event) that additionally incorporates gender, age (binned into 4 categories according to quartiles) and SOFA score at ICU admission (binned into 4 categories according to quartiles). As a reference: the concordance index for a Cox model including only gender, age and SOFA score at ICU admission (excl. AKI criteria) equalled 0.640.

^5^ Estimated by an extended Fine-Gray Cox model (that treats ICU discharge as a competing event)
