# Supplementary material for: The importance of the urinary output criterion for the detection and prognostic meaning of AKI
Source: Sci Rep. 2021 May 27;11:11089. doi: 10.1038/s41598-021-90646-0 (PMC8159993; doi:10.1038/s41598-021-90646-0)
Supplement: Supplementary file 4 — Supplementary Information 4. [file 41598_2021_90646_MOESM4_ESM.docx]

**Supplementary Table 3: Comparison of incidence rates and hazard ratios when entering AKI criteria as time-fixed vs time-varying covariates in a Cox model (sensitivity analysis to illustrate immortal time bias)**

| KDIGO stage≥2 criterion | Incidence rate of ICU mortality  (estimated number of ICU deaths per 1,000 patient days at risk) | | Incidence rate ratio of ICU mortality^1^  (95% CI) | Unadjusted cause-specific hazard ratio of ICU mortality^2^ (95% CI) | Naïve^3^ incidence rate of ICU mortality (estimated number of ICU deaths  per 1,000 patient days at risk) | | Naïve^3^ incidence rate ratio of ICU mortality^1^  (95% CI) | Naïve unadjusted cause-specific hazard ratio of ICU mortality^4^ (95% CI) |
| --- | --- | --- | --- | --- | --- | --- | --- | --- |
|  | Patient days  with AKI | Patient days without AKI |  |  | Patient days with AKI | Patient days  without AKI |  |  |
| SCrea | 31.3 | 16.1 | 1.94 (1.71-2.20) | 2.11 (1.85-2.42) | 26.5 | 17.1 | 1.55 (1.37-1.76) | 1.62 (1.42-1.84) |
| UO-1 | 27.2 | 12.6 | 2.16 (1.90-2.46) | 3.00 (2.55-3.54) | 21.6 | 16.6 | 1.30 (1.14-1.48) | 1.34 (1.17-1.53) |
| UO-2 | 36.6 | 14.1 | 2.59 (2.29-2.92) | 3.21 (2.79-3.69) | 27.0 | 16.0 | 1.69 (1.50-1.91) | 1.80 (1.59-2.05) |
| SCrea-UO-1 | 26.5 | 12.1 | 2.19 (1.92-2.51) | 2.85 (2.43-3.34) | 21.7 | 16.0 | 1.36 (1.19-1.55) | 1.40 (1.22-1.61) |
| SCrea-UO-2 | 32.8 | 13.2 | 2.48 (2.20-2.81) | 2.93 (2.57-3.35) | 26.2 | 15.0 | 1.75 (1.55-1.97) | 1.85 (1.63-2.10) |

***SCrea****: serum creatinine >4.0 mg/dl or >2x baseline, where baseline = SCrea-1 whenever available, otherwise SCrea-2, or SCrea-3 (when neither SCrea-1 nor SCrea-2 are available) with* ***Screa-1*** *defined as baseline Screa measurement as manually entered in ICIS by the treating physician at ICU admission;* ***Screa-2*** *defined as the lowest pre-ICU measurement up to 365 days before ICU admission as extracted from the lab information system;* ***Screa-3*** *defined as a back-calculated baseline Screa using the simplified 4-variable Modification of Diet in Renal Disease (MDRD) Study equation assuming an estimated glomerular filtration rate (eGFR) of 75 ml/min/1.73 m^2^ for every patient^30^;* ***UO-1:*** *total* *UO during the last 12-hour period was ≤ 6 ml/kg;* ***UO-2:*** *total* *UO during each of the last 12 consecutive 1-hour periods was ≤ 0.5 ml/kg;* ***SCrea-UO-1****: AKI stage≥2 according to either the SCrea criterion or the UO-1 criterion;* ***SCrea-UO-2****: AKI stage≥2 according to either the SCrea criterion or the UO-2 criterion.*

^1^ Incidence rate ratios only approximate (cause-specific) hazard ratios when the survival distributions in each group both follow an exponential distribution.

^2^ Estimated by an extended Cox model with AKI criterion entered as a time-varying covariate (and that treats ICU discharge as a censoring event)

^3^ Naïve incidence rates and rate ratios attribute corresponding to treating AKI criteria as time-fixed covariates. These estimates erroneously apportion patient days without/before AKI in AKI diagnosed patients to patient days with AKI.

^4^ Estimated by a Cox model with AKI criterion entered as a time-fixed covariate (and that treats ICU discharge as a censoring event)

Results in this table illustrate that a naïve analytical approach that fails to account for the timing of AKI diagnosis (entering it as a time-fixed covariate), consistently underestimates relative hazards associated with different AKI criteria. Importantly, due to the time-dependent nature of the UO criteria, the downward bias was most pronounced for relative hazards associated with the UO criteria. This may have invalidated the results of other studies that assessed the association between different AKI criteria and adverse outcomes.
