# Supplementary material for: The importance of the urinary output criterion for the detection and prognostic meaning of AKI
Source: Sci Rep. 2021 May 27;11:11089. doi: 10.1038/s41598-021-90646-0 (PMC8159993; doi:10.1038/s41598-021-90646-0)
Supplement: Supplementary file 5 — Supplementary Information 5. [file 41598_2021_90646_MOESM5_ESM.docx]

**Supplementary Table 4: Percentage of missing data for evaluation of the criteria and its impact on incidence of AKI**

[Trek de aandacht van uw lezer met een veelzeggend citaat uit het document of gebruik deze ruimte om een belangrijk punt te benadrukken. Sleep dit tekstvak als u het ergens anders op de pagina wilt plaatsen.]

|  | **% patients with insufficient data available to evaluate criterion** | **Incidence of AKI (%) in patients with sufficient data available to evaluate criterion** | **Incidence of AKI (%) in overall cohort (assuming AKI absent in case of insufficient data)** |
| --- | --- | --- | --- |
| *SCrea-1* | 20.0 | 14.3 | 11.4 |
| *SCrea-2* | 17.5 | 15.8 | 13.0 |
| *SCrea-3* | 1.7 | 16.8 | 16.5 |
| *SCrea-4* | 23.7 | 14.2 | 10.8 |
| *SCrea-5* | 1.7 | 9.7 | 9.5 |
| SCrea | 1.7 | 13.5 | 13.2 |
| UO-1 | 1.5 | 34.8 | 34.3 |
| UO-2 | 1.5 | 14.4 | 14.2 |
| SCrea-UO-1 | 2.8 | 39.8 | 38.7 |
| SCrea-UO-2 | 2.9 | 21.5 | 20.9 |

***Screa-1*** *SCrea >4.0 mg/dl or >2x baseline as manually entered in ICIS by the treating physician at ICU admission;* ***Screa-2*** *SCrea >4.0 mg/dl or >2x baseline defined as lowest pre-ICU measurement up to 365 days before ICU admission as extracted from the lab information system;* ***Screa-3*** *SCrea >4.0 mg/dl or >2x back-calculated baseline calculated using the simplified 4-variable Modification of Diet in Renal Disease (MDRD) Study equation assuming an estimated glomerular filtration rate (eGFR) of 75 ml/min/1.73 m^2^ for every patient;* ***Screa-4*** *SCrea >4.0 mg/dl or >2x baseline defined as lowest pre-ICU measurement of the current hospitalization as extracted from the lab information system****; Screa-5*** *SCrea >4.0 mg/dl or >2x baseline defined as the first measurement taken since ICU admission as extracted from the lab information system;* ***SCrea****: serum creatinine >4.0 mg/dl or >2x baseline, where baseline corresponds to that defined in SCrea-1 whenever available, otherwise SCrea-2, or SCrea-3 (when neither SCrea-1 nor SCrea-2 are available);* ***UO-1:*** *total* *UO during the last 12-hour period was ≤ 6 ml/kg;* ***UO-2:*** *total* *UO during each of the last 12 consecutive 1-hour periods was ≤ 0.5 ml/kg;* ***SCrea-UO-1****: AKI stage≥2 according to either the SCrea criterion or the UO-1 criterion;* ***SCrea-UO-2****: AKI stage≥2 according to either the SCrea criterion or the UO-2 criterion.*
