# Supplementary material for: The importance of the urinary output criterion for the detection and prognostic meaning of AKI
Source: Sci Rep. 2021 May 27;11:11089. doi: 10.1038/s41598-021-90646-0 (PMC8159993; doi:10.1038/s41598-021-90646-0)
Supplement: Supplementary file 6 — Supplementary Information 6. [file 41598_2021_90646_MOESM6_ESM.docx]

**Supplementary Figure 4: Venn-diagram illustrating the distribution and overlap between AKI diagnosis according to different choices of baseline for the SCrea AKI criterion**

[Trek de aandacht van uw lezer met een veelzeggend citaat uit het document of gebruik deze ruimte om een belangrijk punt te benadrukken. Sleep dit tekstvak als u het ergens anders op de pagina wilt plaatsen.]

*
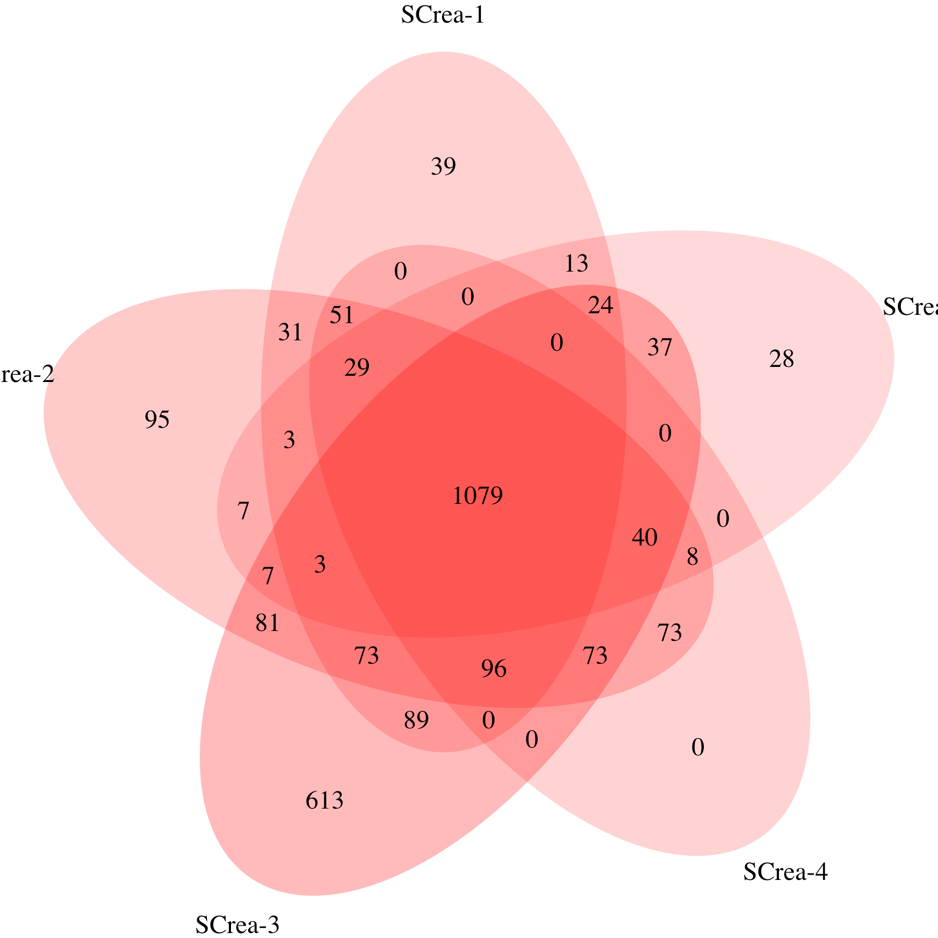
*

***Screa-1*** *SCrea >4.0 mg/dl or >2x baseline as manually entered in ICIS by the treating physician at ICU admission;* ***Screa-2*** *SCrea >4.0 mg/dl or >2x baseline defined as lowest pre-ICU measurement up to 365 days before ICU admission as extracted from the lab information system;* ***Screa-3*** *SCrea >4.0 mg/dl or >2x back-calculated baseline calculated using the simplified 4-variable Modification of Diet in Renal Disease (MDRD) Study equation assuming an estimated glomerular filtration rate (eGFR) of 75 ml/min/1.73 m^2^ for every patient;* ***Screa-4*** *SCrea >4.0 mg/dl or >2x baseline defined as lowest pre-ICU measurement of the current hospitalization as extracted from the lab information system****; Screa-5*** *SCrea >4.0 mg/dl or >2x baseline defined as the first measurement taken since ICU admission as extracted from the lab information system*
